# Examining predictors of reduction in drinking risk-level among severe-risk trauma patients following a brief counseling intervention

**DOI:** 10.1186/1940-0640-8-S1-A50

**Published:** 2013-09-04

**Authors:** Regina R Moro, Laura Veach, Preston Miller, Mary Claire O’Brien

**Affiliations:** 1 Counseling Department, Barry University, Miami Shores, FL, USA; 2 Wake Forest School of Medicine, Winston-Salem, NC, USA; 3 Department of Counseling, University of North Carolina at Charlotte, Charlotte, NC, USA

## 

Significant attention has been given to alcohol screening and brief intervention, with the majority of studies focused on the risky drinking population. The more severe-risk population has been excluded purposely although there is limited evidence as to why this has been established as an acceptable procedure. There have been numerous claims made that the more severe-risk drinker would not respond well to brief interventions, however these claims are not made on the basis of empirical research, only general consensus. The purpose of this study was to identify potential predictors of alcohol screening and brief counseling intervention outcomes for severe-risk drinkers. Specifically, age, gender, race, blood alcohol level, counseling intervention type, injury mechanism, and the constructs of the AUDIT assessment use were examined to see whether the variables were able to predict reduction to low-risk levels among severe-risk participants. The current study was a retrospective analysis of a larger randomized clinical trial. A total of 101 participants were identified as severe-risk from the large study (30.3%). All variables were gathered from participant self-report at baseline. Multivariate logistic regressions were conducted to analyze the data. The results of the analysis highlighted that over two-thirds (67.3%) of the participants reduced from severe-risk (AUDIT >15) to low-risk (AUDIT <8) at six-month follow-up. The average reduction in AUDIT scores was 14 points, representing a large effect size with a Cohen’s *d* value of 1.68. The two models examined in the logistic regression were non-significant, indicating there is little support for the predictors examined within this analysis. These results imply that severe-risk drinkers can benefit from receiving a brief counseling intervention while hospitalized after a traumatic injury. More research focused on the severe-risk drinker is needed.

